# Impact on Sensory Typicity and Consumer Acceptance of the New Regulatory Alcohol Framework for Biologically Aged Sherry Wines: An Industrial-Scale Study

**DOI:** 10.3390/foods15203625

**Published:** 2026-10-12

**Authors:** Lourdes Vega-Espinar, Víctor Palacios, Gustavo Cordero-Bueso, Juan Moreno, Cristina Lasanta

**Affiliations:** 1 Department of Chemical Engineering and Food Technology, University of Cádiz, Agrifood Campus of International Excellence (CeiA3), Wine and Agri-Food Research Institute (IVAGRO), 11510 Cádiz, Spain; lourdes.espinar@uca.es (L.V.-E.); victor.palacios@gm.uca.es (V.P.); 2 Department of Biomedicine, Biotechnology and Public Health (Section of Microbiology), University of Cádiz, Wine and Agri-Food Research Institute (IVAGRO), 11510 Cádiz, Spain; gustavo.cordero@uca.es; 3 Department of Agricultural Chemistry, Edaphology and Microbiology, Marie Curie (C3) and Severo Ochoa (C6) Buildings, Agrifood Campus of International Excellence (CeiA3), University of Córdoba, Ctra, N-IV-A, km396, 14014 Cordoba, Spain; qe1movij@uco.es

**Keywords:** Sherry wine, biological aging, dynamic ‘Solera y Criaderas’ system, ethanol reduction, industrial scale, sensory typicity, consumer acceptance, regulatory framework

## Abstract

The reduction in alcohol content in Protected Designation of Origin (P.D.O.) ‘Jerez-Xérès-Sherry’ biologically aged wines represents a major regulatory and commercial milestone to align these historic products with modern global health trends. This study validates the feasibility of reducing the ethanol content from the traditional 15% *v*/*v* to 14% *v*/*v* through comprehensive industrial-scale monitoring under the traditional dynamic ‘Solera y Criaderas’ aging system the majority method in the region in two distinct wineries, followed by consumer preference testing and a 36-month bottle stability study. Industrial monitoring revealed that a 14% *v*/*v* ethanol environment promotes active flor yeast metabolic activity, as evidenced by sustained ethanol consumption and higher acetaldehyde accumulation, successfully maintaining active biological aging. Furthermore, volatile acidity remained consistently low, confirming the absence of significant microbiological alterations during the process. Hedonic consumer tests (*N* = 557) demonstrated that the 14% *v*/*v* wine is sensorially comparable to the 15% *v*/*v* control but significantly preferred, whereas a further reduction to 13% *v*/*v* reduced sensory balance and overall consumer acceptance. Over 36 months of bottle storage at 20 °C, the 14% *v*/*v* wine maintained high physical stability, preserving its characteristic pale gold color, clarity, and essential biological aging profile (including ’yeast’ and ‘nuts’ descriptors). Minor differences in browning (absorbance at 420 nm) emerged at 24 and 36 months (*p* = 0.0066) but did not impact overall quality. Crucially, the official tasting panel of the OECCA Foundation formally declared all 14% *v*/*v* samples as compliant with P.D.O. organoleptic specifications throughout the entire 3-year study. These findings demonstrate that reducing the alcoholic strength of Fino-style wines to 14% *v*/*v* is technically and commercially viable under traditional dynamic aging conditions, preserving enological heritage while satisfying modern consumer demands.

## 1. Introduction

Wines protected under the ‘Jerez-Xérès-Sherry’ P.D.O. represent an exceptional enological heritage, founded on a tradition dating back to 1100 B.C. during the Phoenician era [1]. Currently, the sector faces a paradigm shift driven by a global decline in wine consumption, which in 2024 reached its lowest levels since 1961 [2]. The Jerez region has been particularly affected; after reaching commercial peaks in the 1970s and 1980s, driven by exports to markets such as the United Kingdom, the Netherlands, and Germany, the sector has experienced a severe and continuous contraction in sales volumes, losing more than half of its market due to shifting global preferences [3]. This trend has generated an increasing demand for products with lower alcoholic strength, perceived as fresher and lighter by new consumers, and aligned with contemporary preferences for healthy lifestyles and responsible consumption [4,5].

In response to this challenge, the European Commission established a regulatory milestone through Implementing Regulations (EU) 2025/1713 and 2025/1714 [6,7], authorizing the modification of the ‘Jerez-Xérès-Sherry’ P.D.O. product specifications. This new legal framework, consolidated at the regional level by the Order of 13 May 2026 (BOJA No. 95) [8], permits for the first time the commercialization of biologically aged wines in the Jerez region with a minimum alcohol content of 14% *v*/*v*. This shift allows the sector to reduce its historical dependence on traditional fortification with wine alcohol (previously fixed at a 15% *v*/*v* threshold), thereby enhancing energy efficiency and reducing the carbon footprint of the production process [9].

Biological aging using the dynamic ‘Solera y Criaderas’ system constitutes one of the most complex and unique enological optimization processes in the world [10]. This technical architecture is organized into scales of American oak (*Quercus alba*) casks. Unlike conventional static aging (vintages), where the lack of replenishment leads to progressive nutrient depletion that can affect flor yeast growth, the dynamic industrial system is defined by periodic operations known as ‘sacas’ (extractions) and ‘rocíos’ (replenishments) (Figure 1).

These movements ensure product homogeneity and introduce fresh nutrients essential for maintaining the metabolic activity of the ‘veil of flor’ a natural biofilm composed mainly of *Saccharomyces cerevisiae* (varieties *beticus*, *cheresiensis*, and *montuliensis*) [11,12,13] and non-*Saccharomyces* yeasts [14,15] that colonize the liquid-air interface in casks maintained with approximately 100 L of headspace [16]. While previous studies successfully validated the feasibility of these findings under controlled conditions, translating these findings to a dynamic system remains a fundamental scientific and technical challenge under the new regulatory framework.

During this period, yeast metabolism drastically modifies the wine matrix; the yeasts consume dissolved oxygen, thereby protecting the medium from direct oxidation, while intensely metabolizing glycerol and organic acids as carbon sources. Furthermore, they synthesize volatile compounds that are decisive for the product’s typicity, most notably the production of acetaldehyde, which is responsible for the pungent aromas defining the character of Sherry wines [17,18]. Traditionally, the structural and cellular viability of this biofilm has been dependent on strict control of the alcoholic strength, regulatorily fixed at approximately 15% *v*/*v*. This threshold ensures the ideal selective pressure for the cellular viability of flor veil strains against opportunistic yeasts and acetic or lactic acid bacteria, while simultaneously guaranteeing the overall physicochemical stability of the wine over the years [19,20].

The organoleptic complexity resulting from biological aging constitutes the core of these wines’ typicity, a factor strictly protected by the current P.D.O. specifications, which require specific ‘yeast’-derived, ‘green apple’, and ‘nuts’ descriptors [8]. While static studies indicate that lower alcohol content can intensify flor metabolism [9,21], it is unknown if the intrinsic dynamism of the Solera system will yield the same effect. Furthermore, research exists on how alcohol reduction itself can impact a wine’s sensory balance [22]. Therefore, a multidimensional evaluation is required: an accredited expert panel to certify technical compliance with D.O. standards, and a consumer study [23,24]. Investigating the hedonic response is essential to determine if the target audience perceives these variations and if a preference exists for lighter profiles. This validation is fundamental to ensure that regulatory innovations translate into a competitive advantage without compromising sensory quality or consumer acceptance.

Consequently, this study aims to comprehensively validate the reduction in ethanol to approximately 14% *v*/*v* under the real-world conditions of the dynamic ‘Solera y Criaderas’ system. An industrial-scale experimental design was implemented over a full operational cycle to contrast previous static results obtained at a smaller scale. This approach determines if the new regulatory flexibility maintains the standards of excellence and typicity that define these historic wines, while opening the possibility of revitalizing the sector with a healthier profile that responds to new global consumption preferences.

## 2. Materials and Methods

### 2.1. Industrial Wineries and Experimental Design

Experimental trials were conducted at an industrial scale in two representative wineries (Winery A and Winery B) located within the ‘Jerez-Xérès-Sherry’ P.D.O. in Spain. In each winery, two parallel dynamic ‘Solera y Criaderas’ systems were monitored simultaneously: a Control system, maintained at the traditional alcoholic strength (~15% *v*/*v*), and a Test system, adjusted to a reduced ethanol content (~14% *v*/*v*). The infrastructure for Winery A involved a total volume of 24,000 L across 48 American oak casks (600 L capacity, filled to ~500 L to facilitate ‘veil of flor’ growth), with 24 casks dedicated to each system structured into three scales of 8 casks each. Winery B managed a total volume of 120,000 L utilizing 240 casks, where 120 casks were assigned to each system and organized into three scales with 40 casks per scale. In both wineries, dynamic aging was managed through traditional ‘saca y rocío’ (extraction and replenishment) operations performed every three months following standard industrial schedules. The temperature ranged in Winery A from 15.23 °C in winter to 24.00 °C in summer and relative humidity from 68.97 to 72.94%; in Winery B the seasonal range was more pronounced, with temperature varying from 14.63 °C in winter to 26.90 °C in summer, and relative humidity ranging from 65.96 to 83.0%.

While nominal target alcohol concentrations were established at 15.0% *v*/*v* for the Control system and 14.0% *v*/*v* for the Test system, full-scale industrial operations in dynamic Criaderas y Solera systems naturally exhibit minor ethanol fluctuations. These variations are driven by the seasonal metabolic consumption of ethanol by the flor veil and the real-world operational timing of saca (extraction) and rocío (replenishment) operations.

### 2.2. Sampling Protocol

The industrial systems were monitored over a full annual operational cycle with sampling points every three months (t = 0, 3, 6, 9, and 12 months). To ensure a representative matrix for routine monitoring, samples were collected from the entire set of casks comprising the ‘solera’ scale in each winery immediately prior to the periodic ‘saca y rocío’ operations. A traditional ‘venencia’ was utilized to draw wine from each individual cask, which was then blended to obtain a homogeneous composite sample, distributed into 75 cL bottles, and immediately transported for analytical and expert sensory tracking. For consumer perception trials, additional wine batches were extracted from several casks within the ‘solera’ scale that met the target alcoholic strengths (15%, 14%, or 13% *v*/*v*). These selected wines were blended and homogenized to ensure representativeness for each specific alcoholic strength prior to being bottled and stored under refrigeration until the scheduled tasting sessions.

### 2.3. Bottle Stability Study

The long-term stability of biologically aged wines was evaluated using a pair of wines from Winery A, comparing the traditional strength (15% *v*/*v* control) against the reduced strength (14% *v*/*v* test) wine. Prior to bottling, both wines were filtered through 1.2 µm filters and bottled manually into 75 cL formats. For each wine type, a total of 18 bottles were prepared (36 bottles in total) and immediately stored in thermostated cabinets under controlled temperature conditions of 20 °C for a 36-month monitoring period. Periodic analytical and organoleptic tracking was performed at 0, 3, 6, 12, 24, and 36 months. At each sampling point across the 6 temporal intervals (0, 3, 6, 12, 24, and 36 months), 3 bottles of each wine type were withdrawn and analyzed to ensure representative and statistically robust results.

### 2.4. Physicochemical Analysis

Standard enological parameters (alcohol content, total acidity, and pH) were determined according to OIV official methods [25]. Volatile acidity was measured via distillation followed by titration according to the García-Tena method [26]. Spectrophotometric measurements were performed using a UV-Vis Genesys™ 10 system (Thermo Fisher Scientific Inc., Waltham, MA, USA) with quartz cuvettes, where the Total Polyphenol Index (TPI) was determined at 280 nm (1:10 dilution, 1 mm path length) and color evolution was assessed at 420 nm (10 mm path length). All samples were pre-filtered through 0.45 µm syringe filters.

### 2.5. Expert Sensory Analysis

Sensory evaluations were conducted by a panel of eight expert judges at the OECCA Foundation, an ENAC-accredited product certification body under the requirements of UNE-EN ISO/IEC 17025 [27] for wines protected under the ‘Jerez-Xérès-Sherry’ P.D.O. This panel, consisting of eight expert evaluators performed the sensory assessment in a controlled environmental condition using individual sensory booths to minimize external interference in accordance with ISO 8589 [28], and panelist were selected and trained following ISO 8586 [29]. During each session, 30 mL wine samples were served in standardized wine-tasting glasses [30], at a controlled temperature of 20 °C. The panel evaluated the sensory attributes across the phases described in Table 1. Results for each tasting session integrated the specific values for every parameter according to the evaluation scales detailed in Table 1, followed by a formal declaration of conformity or non-conformity with the organoleptic standards required by the P.D.O. product specifications.

For each olfactory attribute, the detection frequency (%) was calculated at each sampling point (t = 0, 3, 6, 9, and 12 months) based on panel responses. To evaluate the overall profile and sensory stability over the annual cycle, the mean detection frequency (%) and its corresponding standard deviation across the 5 temporal sampling points (N = 5) were computed for each system and winery.

### 2.6. Consumer Sensory Evaluation

Market acceptance and the sensory impact of alcohol reduction were assessed through paired comparison tests strictly following the ISO 5495 guidelines [31]. Wines at 14% and 13% *v*/*v* were selected directly from individual oak casks that naturally exhibited these specific alcoholic strengths. The study involved a heterogeneous group of participants, comprising wine sector professionals as well as regular consumers and enthusiasts of Jerez wines, recruited across different sessions. This broad recruitment approach ensured a representative cross-section of the target consumer market for Jerez wines. The 15% vs. 14% *v*/*v* comparison was extensively tested (N = 451) as the primary target reduction level, whereas the 15% vs. 13% *v*/*v* comparison was carried out in specific sessions (N = 106) as an exploratory evaluation to check whether consumers could perceive an even greater reduction. For these sessions, wine samples from the same winery were served at temperatures between 8 and 10 °C in standardized tasting glasses. Participants performed two forced-choice sensory tasks: a hedonic preference test to determine significant inclinations toward a specific strength and an intensity perception test to identify which wine possessed the higher alcohol content, thereby evaluating the perceptibility of the reduction.

### 2.7. Statistical Analysis

Comparative statistical analysis was performed using GraphPad Prism v11.0 (GraphPad Software, San Diego, CA, USA). One-way analysis of variance (ANOVA) followed by Šidák’s post-hoc test was used to determine significant differences (*p* < 0.05) in physicochemical parameters. Multivariate statistical processing of sensory data, including Principal Component Analysis (PCA), Hierarchical Cluster Analysis (HCA), and Binomial Tests, was executed using IBM SPSS Statistics v29 (International Business Machines Corporation, Armonk, NY, USA). Consumer trial data were subjected to the exact binomial test and the standard normal approximation to define critical regions of significance according to international standards.

## 3. Results and Discussion

### 3.1. Physicochemical Parameters

Table 2 and Table 3 summarize the evolution of the primary physicochemical parameters measured in the laboratory and monitored in the ‘solera’ scale of the dynamic aging systems in both facilities (Winery A and Winery B) over a full annual cycle (t = 0, 3, 6, 9, and 12 months).

Regarding ethanol content, significant differences (*p* < 0.0001) were observed between control and test wines across all sampling points in both facilities. At the onset of the study (t = 0), control casks presented initial alcoholic strengths of 14.67% *v*/*v* (Winery A) and 15.27% *v*/*v* (Winery B), while test casks exhibited baseline values of 14.12% *v*/*v* and 14.11% *v*/*v*, respectively. In Winery A, alcohol concentration remained constant throughout the year due to the quarterly ‘saca y rocío’ operations, maintaining a stable mean gap of 0.38% *v*/*v* between systems. Conversely, the control system in Winery B showed a 4% decrease over time (annual average 14.90% *v*/*v*), while its test system remained stable at 14.11% *v*/*v*. This resulted in an average difference of 0.79% *v*/*v* in Winery B, nearly double that of Winery A. Despite minor operational deviations from nominal target values under real cellar operations, a statistically significant ethanol gradient (*p* < 0.0001) was successfully and continuously maintained between the Control (~14.7–15.3% *v*/*v*) and Test (~14.0–14.4% *v*/*v*) systems across all sampling stages in both wineries throughout the annual cycle.

Volatile acidity is a critical control parameter for biological aging, as acetic acid serves as a substrate that can be metabolized by flor veil yeast during the process [32]. Furthermore, this compound can be synthesized through the metabolism of lactic acid bacteria, and especially by *Brettanomyces* and acetic acid bacteria [33]. As detailed in Table 2 and Table 3, no significant differences were found between the control and test systems in either winery, with values remaining within a narrow range of 0.12 to 0.22 g L^−1^. These results indicate that no significant microbial contamination occurred, which is likely attributable to the periodic ‘saca y rocío’ operations that maintain a much more stable flor veil compared to static systems. This finding is particularly relevant as previous studies on static biological aging demonstrated that alcohol reduction increased the probability of volatile acidity elevation and microbial proliferation [9]. Consequently, the dynamic system serves as an effective tool for microbiological control and safety when conducting biological aging at a reduced alcoholic strength of ~14% *v*/*v*.

Regarding total acidity in Winery A, the control system presented higher values than the test system during the initial stages (t = 0 and 3 months; *p* < 0.005), with no significant differences observed from month 6 onwards. In Winery B, no differences in acidity were found between systems except at month 9 (*p* < 0.005).

Absorbance at 420 nm (Abs 420 nm) and the TPI were utilized to assess the degree of oxidation or browning and the concentration of polyphenols during biological aging [34,35]. Winery B exhibited higher Abs 420 and TPI values than Winery A, resulting in a greater degree of browning and polyphenol concentration. This development is influenced by the respiratory activity of the flor yeast, which regulates the redox potential of the wine [36]. Nevertheless, no significant differences were observed between the control and test systems for either parameter in the studied wineries.

The temporal evolution followed a similar pattern in both wineries. During the first three months (April to June), Abs 420 remained constant. However, between 3 and 6 months (July to October), coinciding with summer temperature peaks, a significant increase in browning was recorded. This indicates a higher dissolution of oxygen in the wine due to reduced flor yeast respiratory activity. In Winery B, the higher TPI resulted in a more pronounced browning effect.

Conversely, between 6 and 12 months, corresponding to autumn, winter, and spring, browning generally decreased though to a lesser extent in Winery B due to more intense yeast respiratory activity that restored a reducing environment within the wine [37].

### 3.2. Sensory Analysis

Sensory assessments were carried out by the expert panel of the OECCA Foundation, the official body habitually responsible for the organoleptic certification of wines under the ‘Jerez-Xérès-Sherry’ P.D.O. This evaluation integrated visual, olfactory, and gustatory assessments of all samples from the control and test systems at t = 0, 3, 6, 9, and 12 months.

#### 3.2.1. Visual Evaluation

Complete color homogeneity was recorded during the visual assessment. The panel unanimously classified the color of all samples, regardless of winery, sampling time, or ethanol level, with a score ≤ 3.5 , corresponding to the descriptor ‘pale gold’ on the structured chromatic scale (Table 1). All wines remained clear and free of turbidity throughout the study. These results indicate that the expert tasters were unable to perceive the analytical variations in browning (Abs 420 nm) recorded during the experimental phase, confirming that the alcohol reduction to 14% *v*/*v* did not impact the visual typicity or commercial standards of the product.

#### 3.2.2. Olfactory Evaluation

The olfactory results, expressed as detection percentages (frequency of detection) for the 11 attributes evaluated, are detailed in Tables S1 and S2 of the Supplementary Material. Three attributes ‘raisins’, ‘honey’, and ‘caramel’ were not detected in any sample (0% detection), confirming the absence of oxidative or sweet descriptors untypical of biological aging. Average values for the perceived attributes across both wineries are represented in Figure 2, where ’yeast’, ’apple’, and ’nuts’ emerged as the dominant descriptors.

When analyzing the interaction between alcoholic strength and these descriptors, a divergent behavior was observed between wineries. In Winery A, the test system (~14% *v*/*v*) exhibited higher average scores for ‘green apple’ and ’nuts’ compared to the control, while the control system (~15% *v*/*v*) excelled in ’white flowers’ and ’yeast’. In Winery B, the control system maintained higher scores for ’nuts’ and ’white flowers’, while test wines did not show higher frequencies than the control in any major attribute, presenting a 16.2% lower frequency for ’white flowers’. Notably, ’white flowers’ were the only attribute that followed a consistent trend in both wineries, with higher perception in the control systems.

A significant finding was that Winery A showed greater sensory divergence between its systems than Winery B, despite Winery B presenting a more pronounced difference in ethanol content between control and test wines (Table 2 and Table 3). This paradox indicates that the absolute difference in alcoholic strength between 15% *v*/*v* and 14% *v*/*v* is not the primary determinant for variations in the olfactory profile of biologically aged wines. Instead, these results suggest that the winery factor and its specific microbiota are more decisive. In Winery A, the sensory variations observed at lower alcohol levels are likely attributable to alterations in the dominant yeast strains or an intensification of cellular metabolism, a phenomenon previously reported in studies of static or vintage aging systems at reduced strength [21].

The temporal evolution of these sensory profiles is shown in Figure 2. Regarding variability over time, Winery A showed higher standard deviations for ’white flowers’ and ’apple’, while Winery B exhibited more stable profiles for most attributes. This confirms that biological aging attributes in Winery B are more consistent and less dependent on alcoholic strength than in Winery A, where seasonal variations are more pronounced at 14% *v*/*v*.

#### 3.2.3. Multivariate Statistical Analysis

Principal component analysis (PCA) was applied to the olfactory attributes to evaluate the distribution of the samples, extracting two factors that accounted for 62.38% of the total cumulative variance (Table 4).

Principal component analysis (PCA) was applied to the olfactory attributes to evaluate the distribution of the samples, extracting two factors that accounted for 62.38% of the total cumulative variance (Table 4). F1 explained 35.16% of the variance and was positively correlated with ’white flowers’ (0.802) and ’nuts’ (0.712), while showing a strong negative correlation with the ’yeast’ descriptor (−0.713). This factor primarily defines the contrast between the floral/oxidative evolution of the wine and the characteristic ’yeast’-derived aromas, typically occurring when flor veil activity is attenuated. F2 accounted for 27.22% of the variance and showed a strong positive correlation with ‘green apple’ (0.884), an attribute closely associated with acetaldehyde content and the metabolic vigor of the flor yeast.

The distribution of samples in the PCA biplot (Figure 3) revealed a clear seasonal influence on the sensory profile. Most control and test samples from t = 3 and 6 months (late spring to early autumn) were positioned in the positive region of F1. This timeframe coincides with peak winery temperatures, which can reduce flor yeast respiratory activity and enhance the perception of oxidative notes to the detriment of the ’yeast’ descriptor. Conversely, samples from t = 0, 9, and 12 months (late autumn to early spring) were located in the negative region of F1, reflecting cooler periods that favor ’yeast’ growth and the predominance of the ‘yeast’ or bakery descriptor.

Regarding F2, positive values were observed for samples at t = 0, 9, and 12 months, indicating active aerobic metabolism and acetaldehyde accumulation. Notably, the highest F2 scores were recorded in test barrels (e.g., Winery A at 9 months and Winery B at 0 months), which aligns with reports suggesting that biological aging at reduced alcoholic strength (~14% *v*/*v*) can enhance acetaldehyde synthesis [9].

Hierarchical cluster analysis (HCA) using Ward’s method and Euclidean distance (Figure 4) confirmed these groupings through three distinct segments. Cluster 1 primarily grouped low-alcohol test samples (TA t = 0 to 12 months) and Winery A control at 9 months, corresponding to stages of high metabolic activity characterized by acetaldehyde-driven profiles and minimal oxidative evolution. Cluster 2 grouped intermediate samples showing greater complexity between acetaldehyde and oxidative descriptors. Cluster 3 was dominated by control system samples at advanced stages, where the oxidative character was most tangible. Despite these groupings, the PCA was unable to globally discriminate between control and test wines or between wineries, demonstrating that the reduction to 14% *v*/*v* does not induce significant shifts in the characteristic aromatic profile required by the P.D.O.

#### 3.2.4. Gustatory Evaluation

The gustatory attributes: acidity, sweetness, bitterness, and salinity were evaluated using a 3-point intensity scale, with results summarized in Table 5. Sweetness was rated as 0 (absence) in all samples, confirming their status as dry wines in accordance with P.D.O. requirements.

For Winery A, both control and test systems maintained constant values for salinity and bitterness at a low intensity (level 1) throughout the trial. However, perceived acidity in the control system fluctuated, with higher values at the onset (t = 0) followed by a decrease at 3 months and a subsequent rise at 9 and 12 months, coinciding with periods of high yeast metabolic activity. In contrast, the test wine (14% *v*/*v*) showed no variations in perceived acidity across all sampling points.

In Winery B, salinity was consistently rated at level 1. Bitterness showed a transient absence at t = 0 for the test wine before stabilizing at level 1, mirroring the control system for the remainder of the study. Regarding perceived acidity, the control wine showed an increase at 6 months followed by a decrease, while the test wine remained stable until a slight increase was noted at month 12.

Most significantly, the official report issued by the expert panel formally declared all evaluated wines, regardless of winery or alcoholic strength, as compliant with the organoleptic specifications established in the P.D.O. product specifications. This certification confirms that reducing the ethanol content to 14% *v*/*v* does not compromise the traditional typicity of these wines, ensuring their suitability for commercialization under the current regulatory framework.

### 3.3. Consumer Sensory Evaluation

The results of the consumer paired comparison tests (ISO 5495) are summarized in Table 6. This section assesses the hedonic preference and the discriminative ability of consumers regarding alcohol reduction to validate the new regulatory limit (14% *v*/*v*) and explore lower thresholds (13% *v*/*v*).

#### 3.3.1. Hedonic Preference and Regulatory Validation

In the comparison between 15% *v*/*v* and 14% *v*/*v* wines, a significant preference (*p* = 0.024) was recorded for the lower-alcohol sample, with 55.44% of consumers choosing the 14% *v*/*v* wine. This statistical significance is robustly supported by the high power of the test, derived from a large sample size (*N* = 451). This preference can be linked to the findings of the expert panel, which noted that the 14% *v*/*v* wines particularly in Winery A exhibited higher frequencies of fresh descriptors such as ‘green apple’ (18.4% higher than the control) and ‘nuts’. Furthermore, the expert panel identified a more stable perceived acidity in the 14% *v*/*v* wines throughout the study, contributing to a more balanced palate. These factors suggest that a moderate reduction in alcohol enhances the aromatic freshness and structural balance, aligning with current market trends for lighter products. These findings validate the regulatory shift, suggesting that the approved reduction not only maintains but may enhance consumer appeal.

In contrast, the exploratory trial between 15% *v*/*v* and 13% *v*/*v* showed no significant hedonic preference (*p* = 0.206), indicating that more aggressive reductions may not necessarily improve consumer appeal.

#### 3.3.2. Perceived Alcohol Intensity and Discriminative Thresholds

Sensory intensity trials demonstrated that the 14% *v*/*v* wine is perceived as sensorially comparable to the traditional 15% *v*/*v* control by target consumers. Only 220 correct identifications were recorded (*N* = 451), which is well below the critical threshold of 243 correct identifications required by ISO standards. This indicates that consumer responses follow a random distribution (*p* = 0.638) and are unable to consciously distinguish between the two alcohol concentrations.

However, in the comparison between 15% *v*/*v* and 13% *v*/*v*, 65.10% of participants (69 correct identifications out of 106) correctly identified the sample with higher alcohol. This result was highly significant (*p* = 0.002), exceeding the critical threshold of 62 correct identifications. These data demonstrate that while 14% *v*/*v* remains below the threshold of conscious perception, a reduction to 13% *v*/*v* crosses a sensory limit that makes the change clearly detectable.

#### 3.3.3. Synthesis of the Optimal Alcohol Level

The integration of these findings identifies 14% *v*/*v* as the “sweet spot” for biologically aged wines. At this level, the reduction is invisible in terms of alcoholic power but significantly preferred by consumers due to the enhanced fresh aromatic profile and structural stability validated by the expert panel. Reducing the strength to 13% *v*/*v* crosses the discriminative threshold, which divides consumer opinion and eliminates the significant hedonic advantage observed at the 14% *v*/*v* level.

### 3.4. Bottle Stability and Long-Term Evolution

#### 3.4.1. Chromatic Evolution

At the beginning of the trial, both the control and test wines exhibited highly similar analytical profiles, with no significant differences in their physicochemical parameters except for their target alcoholic strength.

Regarding chromatic characteristics, wine absorbance evolution at 420, 520, and 620 nm during bottle aging was measured, and the results for all three wavelengths are summarized in Table 7. For the 520 and 620 nm wavelengths, the values remained very low (below 0.1) throughout the entire study period and showed no significant differences between samples. In contrast, significant changes were observed in the values at 420 nm, widely used as an indicator of yellow color intensity and browning processes associated with aging and oxidative reactions; it is the standard parameter for assessing color and browning in these wines, providing a clear indication of their oxidative evolution [38,39].

Both samples showed high stability during the first year of storage at 420 nm. Nevertheless, statistically significant differences emerged at 24 months (*p* = 0.0124) and became more pronounced at the end of the study (t = 36 months, *p* = 0.0066). From 24 months onwards, the 14% *v*/*v* wine exhibited significantly higher absorbance levels than those corresponding to the 15% *v*/*v* control. Specifically, the 14% *v*/*v* wine reached an absorbance of 0.252 ± 0.001 (a 25.37% total increase), while the 15% *v*/*v* control recorded a more moderate increase of 16.5% (0.240 ± 0.000). The divergence suggests that a lower ethanol content may slightly influence the solubility of residual oxygen or accelerate the rate of phenolic condensation reactions over time. Despite these spectrophotometric variations at 420 nm, the physical stability of the wines was remarkable.

#### 3.4.2. Sensory Evolution and Compliance

Visual inspections confirmed that all samples remained perfectly clean and bright, with no development of turbidity or appearance of precipitates in any case. Furthermore, color intensity evaluation remained strictly within the ‘pale gold’ range (score ≤ 3.5, across all samples and storage times, according to the structured scale (Table 1). This ensures that the reduction to 14% *v*/*v* complies with the high visual quality standards required by the sector. Taste profile analysis revealed high temporal stability for acidity, salinity, and bitterness across 36 months of bottle aging, with all attributes scoring low intensity values (intensity 1; Table 1). The palate profile was perceived as well balanced, characterized by a complete absence of perceived sweetness with no detectable gustatory defects.

At the time of bottling (t = 0), both the 15% and 14% *v*/*v* wines presented a characteristic biological aging profile, reflected in the high detection frequencies (100% or close to 100%) for traditional descriptors such as ‘green apple’, ’white flowers’, ’nuts’, and ’yeast’ (associated with the characteristic fresh bread dough and bakery notes stemming from biological aging under the flor yeast veil). The sensory profiles were subsequently tracked over 36 months to evaluate how alcohol reduction impacts the longevity of these attributes during bottle storage (Figure 5). In the absence of an active flor veil, the ‘green apple’ and ’white flowers’ attributes showed a natural and parallel decline after 6 months before reaching stabilization at intermediate levels.

The 14% *v*/*v* wine initially exhibited a higher detection frequency (100%) of the ‘yeast’ descriptor compared to the 15% *v*/*v* control. This finding aligns with the results from the industrial monitoring phase, where the 14% *v*/*v* environment sustained active flor yeast metabolism, resulting in a higher accumulation of key metabolites such as acetaldehyde and yeast-derived volatile compounds prior to bottling. During subsequent bottle storage, these yeast-derived notes stabilize, yielding equivalent sensory profiles for both 14% and 15% *v*/*v* wines over time. Regarding the ’nuts’ descriptor, both wines demonstrated remarkable stability and high detection rates throughout the 36-month period. This attribute remained practically identical in both alcohol concentrations for the majority of the study, maintaining very high levels that underscore the robust preservation of the wine’s core identity despite the one-degree reduction in ethanol. The slight increase to a 100% detection rate in the 15% *v*/*v* control at the 36-month mark is attributed to the relative attenuation of other aromatic descriptors over time rather than a significant shift in the nutty character itself, which remained high in both samples throughout the entire study.

The ’vegetable’ descriptor initially exhibited a higher detection frequency in the 15% *v*/*v* wine. Nevertheless, the evolution of this attribute followed a similar pattern for both alcohol strengths, remaining relatively stable for the first year before declining in a parallel manner to reach very low detection frequencies in both wines by the 36-month mark.

The appearance of ’honey’ notes at the 12- and 36-month sampling points served as a common indicator of slow oxidative evolution within the bottle for both systems [40]. Most significantly, the official tasting panel of the OECCA Foundation formally declared all samples at both 15% and 14% *v*/*v* as compliant with the organoleptic standards established in the P.D.O. product specifications throughout the entire 36-month period. This certification confirms that reducing the alcohol content to 14% *v*/*v* is technically viable for long-term commercialization, as the wine successfully maintains its essential typicity and quality during its commercial lifespan.

## 4. Conclusions

Reducing the alcohol content of P.D.O. ‘Jerez-Xérès-Sherry’ biologically aged Fino-style wines from 15% to 14% *v*/*v* is technically, biologically, and commercially feasible. Industrial-scale trials under the conventional dynamic ‘Solera y Criaderas’ system showed that a 14% *v*/*v* environment sustains active flor yeast metabolism and biological aging. Consistently low volatile acidity levels confirmed that the flor veil remained fully functional, preventing bacterial activity despite the reduced alcohol content. Nevertheless, future research is warranted to evaluate the long-term microbial ecology of the flor veil at the strain level across successive dynamic cycles.

Regarding the descriptive sensory characterization, the expert tasting panel confirmed that the 1% *v*/*v* reduction preserves typicity, showing that olfactory differences are primarily driven by the specific winery factor and its resident microbiota rather than ethanol levels. From a market perspective, consumer hedonic testing revealed that the 14% *v*/*v* wine remains sensorially comparable to the traditional 15% *v*/*v* and significantly preferred by consumers, while further reduction to 13% *v*/*v* compromised sensory balance and decreased acceptance.

Furthermore, the 36-month bottle study confirmed the physical and chromatic stability of the 14% *v*/*v* wine, successfully preserving its core biological aging descriptors (’yeast’ and ’nuts’). This was definitively validated by the OECCA Foundation tasting panel, which declared all 14% *v*/*v* samples from both the industrial and bottle phases fully compliant with P.D.O. specifications. Consequently, reducing the strength to 14% *v*/*v* represents a viable strategy to modernize Andalusian viticultural heritage, preserving its strict typicity and quality while satisfying contemporary market demands.

## Figures and Tables

**Figure 1:**
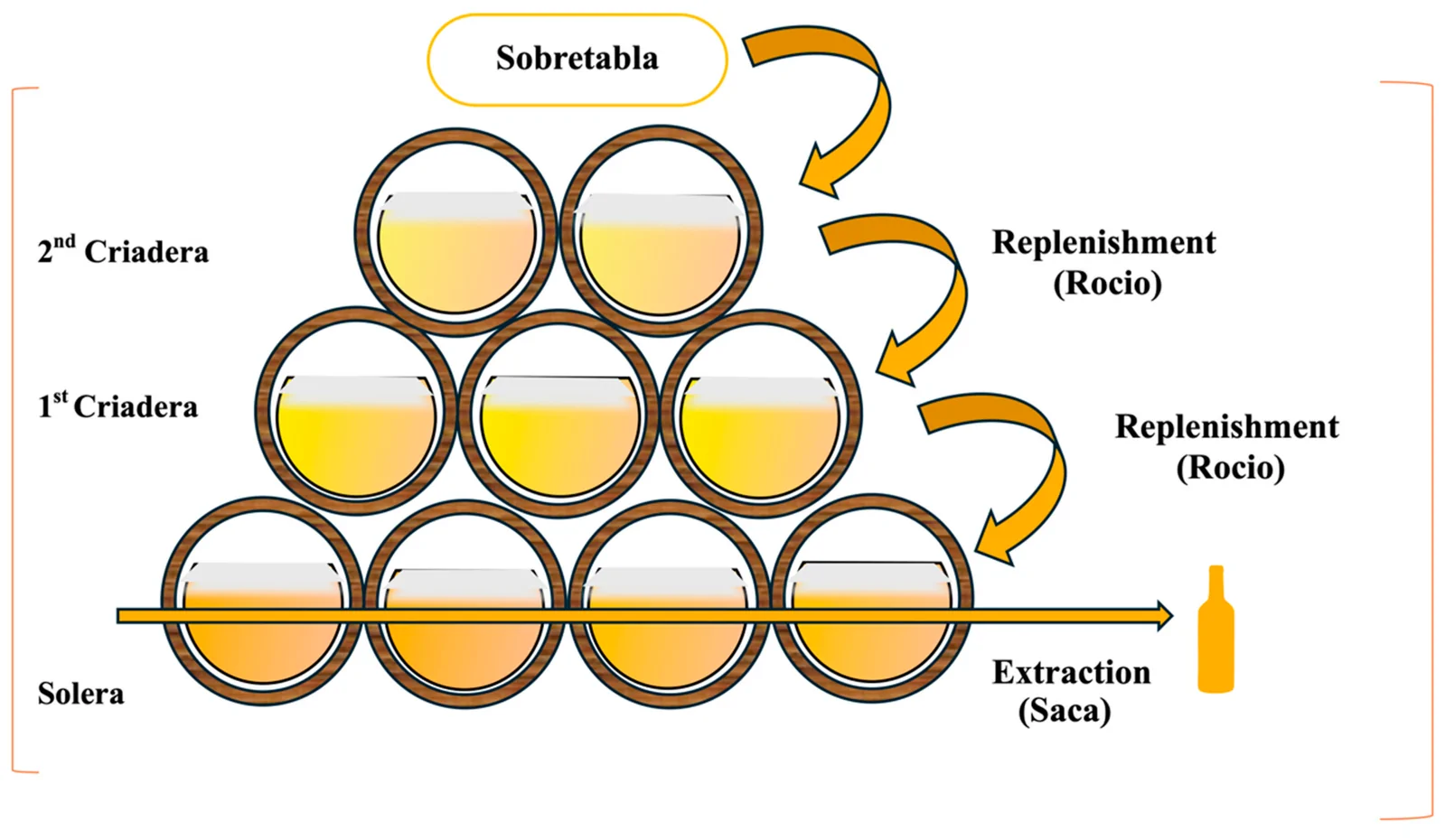
Schematic diagram of the dynamic ‘Solera y Criaderas’ system.

**Figure 2:**
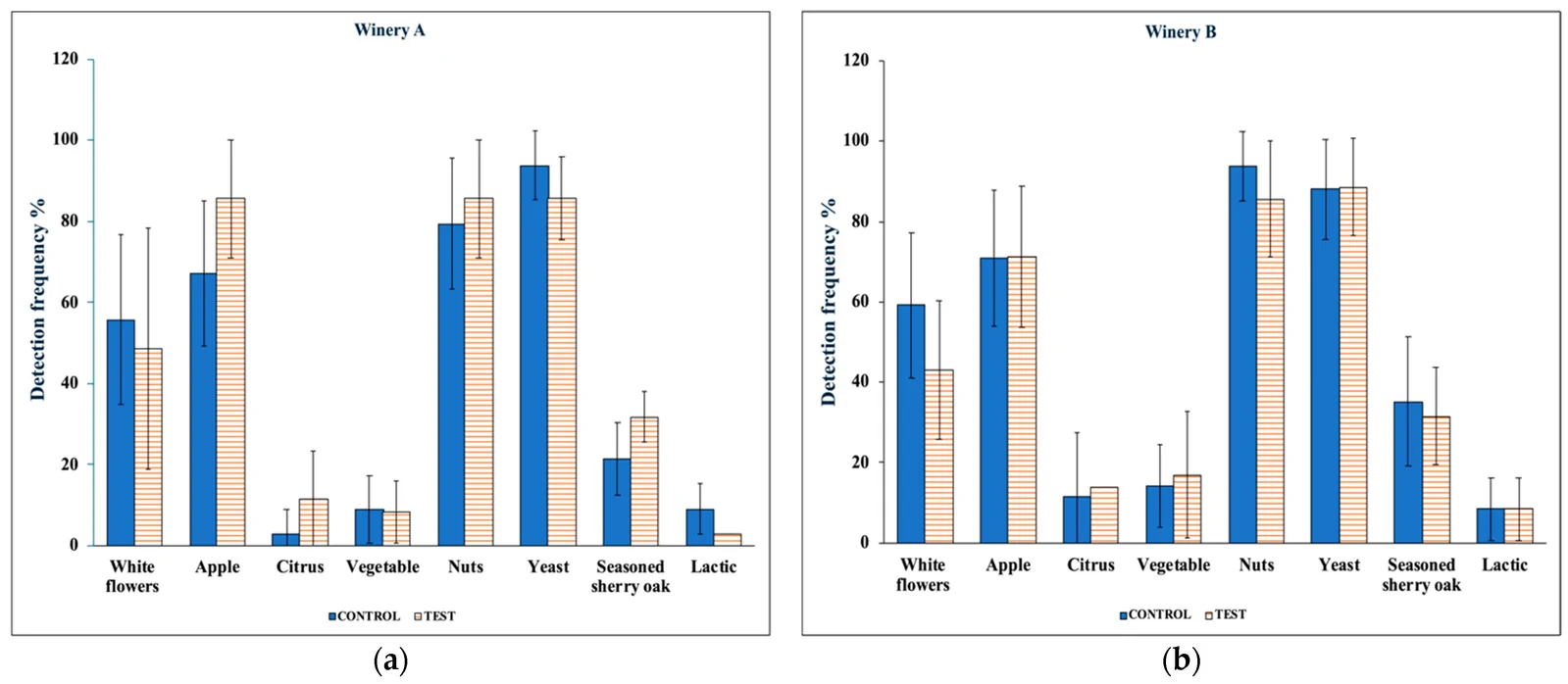
Mean detection frequency (%) and standard deviation (N = 5 sampling points over 12 months) of olfactory sensory attributes for Winery A (**a**) and Winery B (**b**). Solid blue bars represent the Control group (~15% *v*/*v*), and striped bars represent the Test group (~14% *v*/*v*).

**Figure 3:**
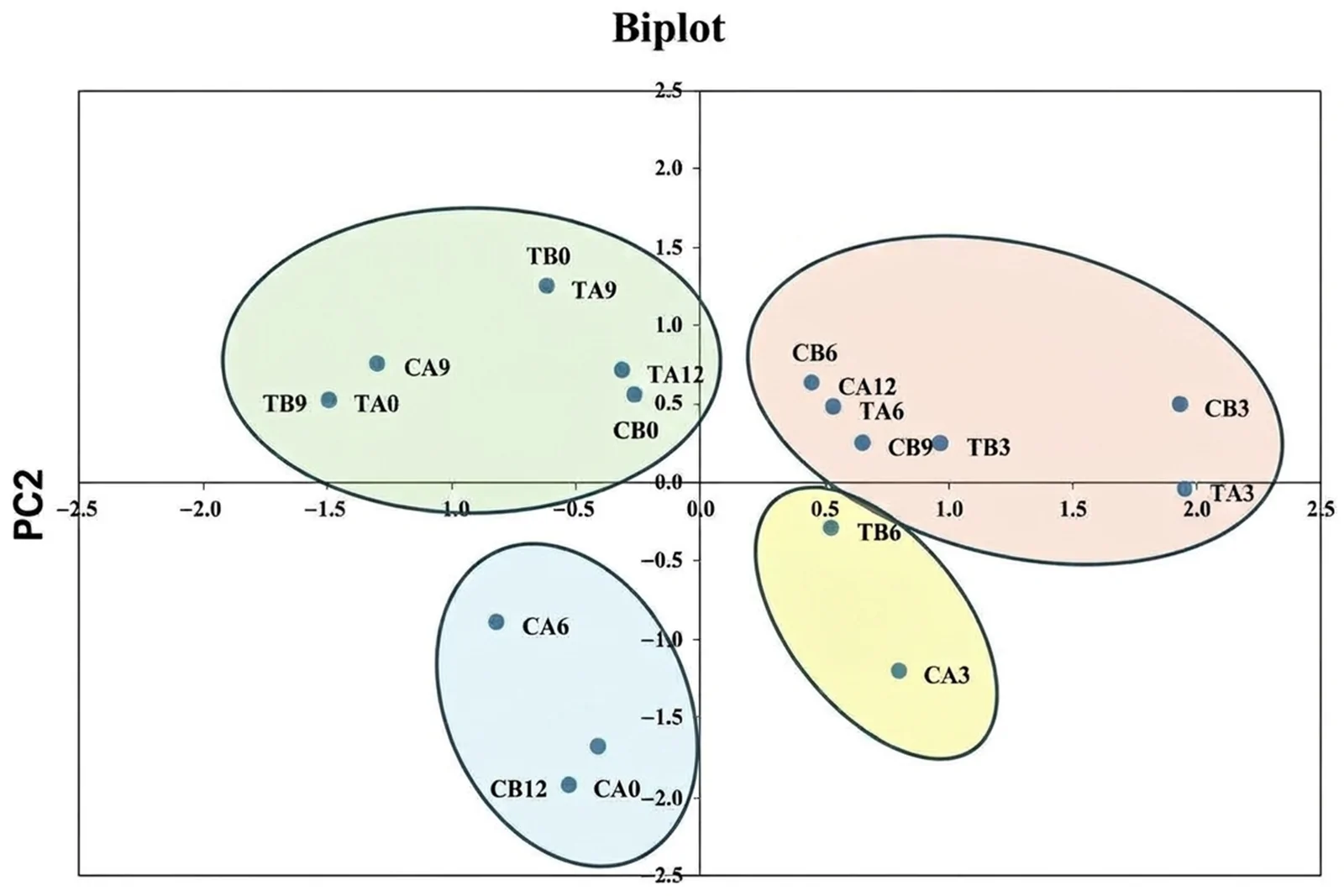
PCA biplot (PC1 vs. PC2) of the sensory attributes. CA = Winery A Control; TA = Winery A Test; CB = Winery B Control; TB = Winery B Test. Numbers indicate aging time (0, 3, 6, 9, 12 months). The shaded ellipses represent the groups.

**Figure 4:**
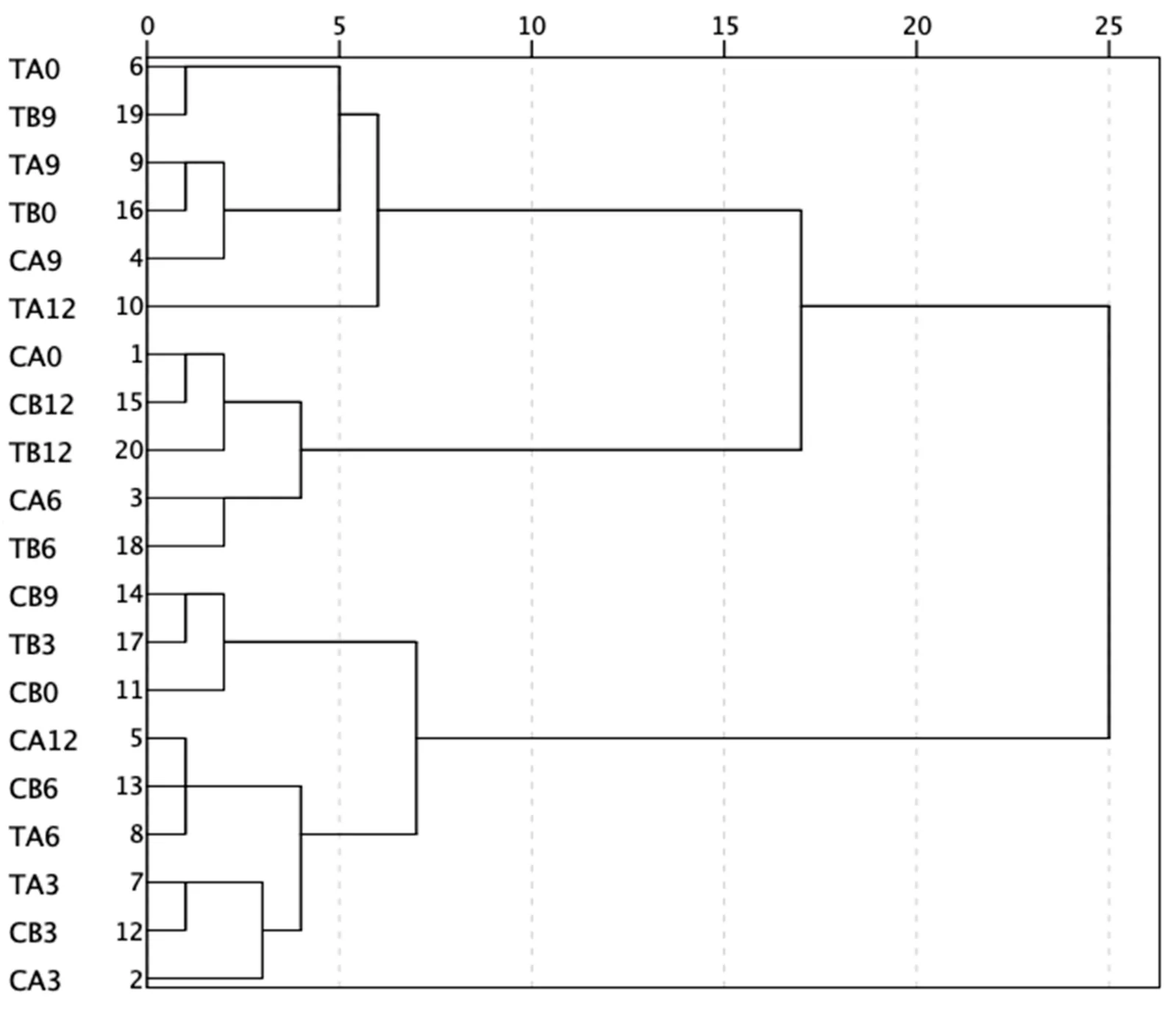
Hierarchical cluster analysis (HCA) dendrogram of wine samples based on sensory attributes using Ward’s method and Euclidean distance. CA = Winery A Control; TA = Winery A Test; CB = Winery B Control; TB = Winery B Test. Numbers indicate aging time (0, 3, 6, 9, 12 months).

**Figure 5:**
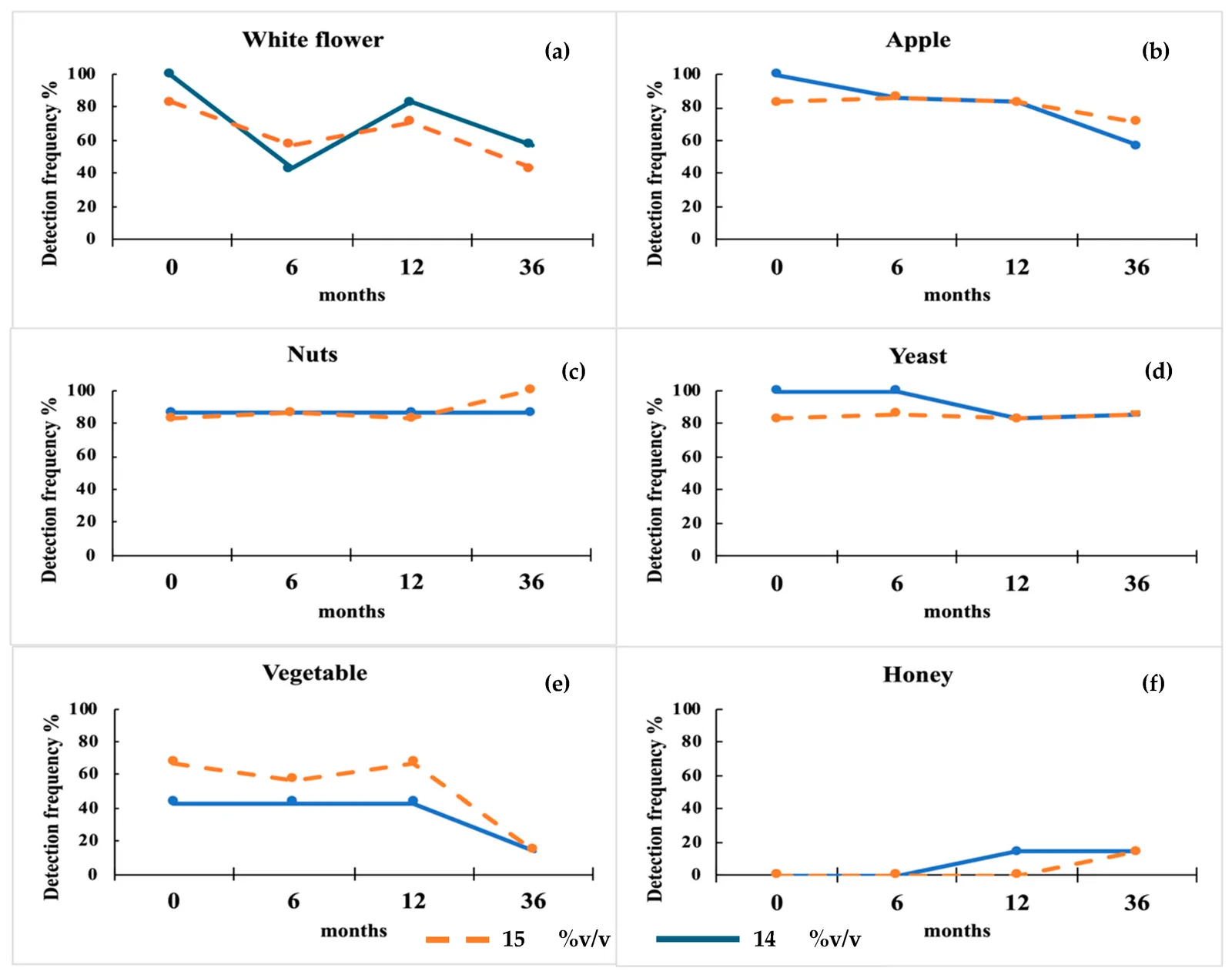
Evolution of sensory attributes (detection frequency %) over time (months): (**a**) white flower; (**b**) apple; (**c**) nuts; (**d**) yeast; (**e**) vegetable; (**f**) honey.

**Table 1:** Sensory attributes, descriptors, and evaluation scales used by the expert tasting panel.

Category	Definition/Descriptor	Evaluation Scale and Intensity Intervals (x)	
Visual phase	Evaluation of the Color Depth (from transparent to dark)	11-point structured discontinued scale	
		x ≤ 0.5	Colorless
		x ≤ 2.5	Straw yellow
		x ≤ 3.5	Pale Gold
		x ≤ 4.5	Gold
		x ≤ 5.5	Amber
		x ≤ 6.5	Dark Amber
		x ≤ 7.5	Chestnut
		x ≤ 8.5	Dark Brown
		x ≤ 9.5	Mahogany
		x ≤ 10.5	Dark Mahogany
		x ≤ 11.0	Ebony
Olfactory phase	Evaluation of 11 Sensory Attributes; Floral (White flowers), Fruit/Citric (Apple, Citrus), Biological Aging (Nuts, Yeast, Seasoned Sherry Oak), Lactic/Sweet (Lactic, Honey, Caramel, Raisin), Vegetable (Vegetable)	Detection Percentage expressed as detection frequency (%) by the panel.	
Gustative phase	Acidity, Sweetness, Bitterness, Salinity	3-point structured discontinued scale	
		0 ≤ x < 0.5	Absence
		0.5 ≤ x < 1.5	Low
		1.5 ≤ x < 2.5	Medium
		2.5 ≤ x < 3	High
Defects	New wood, moisture-earthy/TCA, glue, other defects	3-point structured discontinued scale	
		0 ≤ x < 0.5	Absence
		0.5 ≤ x < 1.5	Low
		1.5 ≤ x < 2.5	Medium
		2.5 ≤ x < 3	High

**Table 2:** Mean and standard deviation of the physicochemical parameters of Winery A.

Winery A	0 Control	0 Test	*p*-Value ^a^	3 Control	3 Test	*p*-Value	6 Control	6 Test	*p*-Value	9 Control	9 Test	*p*-Value	12 Control	12 Test	*p*-Value
pH	3.10 ± 0.035	3.13 ± 0.014	ns	3.07 ± 0.014	3.166 ± 0.028	0.0088	3.11 ± 0.007	3.09 ± 0.000	ns	3.11 ± 0.07	3.12 ± 0.000	ns	3.19 ± 0.000	3.13 ± 0.014	ns
Total acidity (g L^−1^)	4.88 ± 0.060	4.275 ± 0.000	<0.0001	4.44 ± 0.000	3.97 ± 0.128	<0.0001	4.86 ± 0.020	4.80 ± 0.020	ns	5.04 ± 0.021	4.97 ± 0.000	ns	4.94 ± 0.020	4.84 ± 0.000	ns
Volatile acidity (g L^−1^)	0.14 ± 0.035	0.14 ± 0.041	ns	0.12 ± 0.058	0.12 ± 0.041	ns	0.13 ± 0.000	0.15 ± 0.011	ns	0.14 ± 0.011	0.18 ± 0.023	ns	0.15 ± 0.012	0.16 ± 0.000	ns
Ethanol (% *v*/*v*)	14.67 ± 0.042	14.12 ± 0.045	<0.0001	14.40 ± 0.085	13.98 ± 0.090	<0.0001	14.69 ± 0.021	14.48 ± 0.064	0.0057	14.72 ± 0.021	14.39 ± 0.064	<0.0001	14.73 ± 0.085	14.36 ± 0.021	<0.0001
Abs 420 nm	0.164 ± 0.003	0.162 ± 0.002	ns	0.162 ± 0.000	0.163 ± 0.000	ns	0.181 ± 0.001	0.184 ± 0.001	ns	0.160 ± 0.000	0.162 ± 0.001	ns	0.140 ± 0.001	0.145 ± 0.001	ns
Total Polyphenols Index (TPI)	11.20 ± 0.012	11.00 ± 0.000	ns	11.00 ± 0.003	10.6 ± 0.001	ns	10.5 ± 0.004	11.7 ± 0.006	ns	12.1 ± 0.001	12.3 ± 0.001	ns	12.1 ± 0.001	13.0 ± 0.001	ns

^a^ *p*-values adjusted by Šidák’s correction method for multiple comparisons (*p* < 0.05). ns: deviations are not statistically significant.

**Table 3:** Mean and standard deviation of the physicochemical parameters of Winery B.

Winery B	0 Control	0 Test	*p*-Value ^a^	3 Control	3 Test	*p*-Value	6 Control	6 Test	*p*-Value	9 Control	9 Test	*p*-Value	12 Control	12 Test	*p*-Value
pH	3.07 ± 0.015	3.03 ± 0.024	ns	3.03 ± 0.042	3.02 ± 0.085	ns	2.95 ±0.014	2.95 ± 0.007	ns	2.96 ± 0.000	2.98 ± 0.007	ns	3.06 ± 0.007	3.04 ± 0.014	ns
Total acidity (g L^−1^)	4.68 ± 0.060	4.75 ± 0.003	<0.0001	4.52 ± 0.343	4.40 ± 0.141	ns	4.80 ± 0.020	4.62 ± 0.041	ns	4.88 ± 0.083	4.47 ± 0.000	0.0007	4.69 ± 0.061	4.60 ± 0.020	ns
Volatile acidity (g L^−1^)	0.18 ± 0.032	0.16 ± 0.021	ns	0.15 ± 0.046	0.12 ± 0.035	ns	0.23 ± 0.000	0.22 ± 0.012	ns	0.14 ± 0.011	0.09 ± 0.012	ns	0.12 ± 0.012	0.10 ± 0.012	ns
Ethanol (% *v*/*v*)	15.27 ± 0.022	14.1 ± 0.031	<0.0001	15.15 ± 0.000	14.0 ± 0.000	<0.0001	14.73 ± 0.042	14.2 ± 0.049	<0.0001	14.70 ± 0.000	14.1 ± 0.000	<0.0001	14.66 ± 0.064	14.0 ± 0.021	<0.0001
Abs 420 nm	0.184 ± 0.005	0.19 ± 0.002	ns	0.185 ± 0.007	0.19 ± 0.003	ns	0.210 ± 0.000	0.20 ± 0.002	ns	0.204 ± 0.001	0.21 ± 0.001	ns	0.203 ± 0.000	0.20 ± 0.001	ns
Total Polyphenols Index (TPI)	11.90 ± 0.008	11.8 ± 0.006	ns	12.40 ± 0.019	12.9 ± 0.028	ns	14.3 ± 0.006	14.6 ± 0.001	ns	15.0 ± 0.001	16.1 ± 0.001	ns	15.0 ± 0.001	16.3 ± 0.001	ns

^a^ *p*-values adjusted by Šidák’s correction method for multiple comparisons (*p* < 0.05). ns: deviations are not statistically significant.

**Table 4:** Factors extracted from the PCA of the sensory attributes of the wines evaluated (varimax rotation).

	PC1	PC2
White flowers	0.802	0.085
Apple	0.061	0.884
Nuts	0.712	−0.287
Yeast	−0.713	−0.345
Seasoned Sherry Oak	0.008	0.684
Variance %	35.16	27.22

**Table 5:** Mean scores of gustatory sensory attributes (0–3 intensity scale) for Control (15% *v*/*v*) and Test (14% *v*/*v*) wines across dynamic biological aging in Winery A and Winery B during the 12 months of the trial.

**Winery A**	**0 Control**	**0 Test**	**3 Control**	**3 Test**	**6 Control**	**6 Test**	**9 Control**	**9 Test**	**12 Control**	**12 Test**
Acidity	2	1	1	1	1	1	2	1	2	1
Salinity	1	1	1	1	1	1	1	1	1	1
Bitterness	1	1	1	1	1	1	1	1	1	1
Sweetness	0	0	0	0	0	0	0	0	0	0
**Winery B**	**0 Control**	**0 Test**	**3 Control**	**3 Test**	**6 Control**	**6 Test**	**9 Control**	**9 Test**	**12 Control**	**12 Test**
Acidity	1	1	1	1	2	1	1	1	2	2
Salinity	1	1	1	1	1	1	1	1	1	1
Bitterness	1	0	1	1	1	1	1	1	1	1
Sweetness	0	0	0	0	0	0	0	0	0	0

**Table 6:** Results of hedonic tastings and perceived intensity in tastings with consumers.

**Hedonic Preference**	**15% vs. 14%** **(** ***N*** ** = 451)**	**(%)**	**15% vs. 13%** **(** ***N*** ** = 106)**	**(%)**
Choose wine control (15%)	201	44.56	60	56.60
Choose wine test (14% or 13%)	250	55.44	46	43.40
*p*-value *	0.024 (*p* < 0.05)		0.206	
**Perceived Alcohol Intensity**	**15% vs. 14%** **(** ***N*** ** = 451)**	**(%)**	**15% vs. 13%** **(** ***N*** ** = 106)**	**(%)**
Choose wine control (15%)	220	48.78	69	65.10
Choose wine test (14% or 13%)	231	51.22	37	34.90
*p*-value *	0.638		0.002 (*p*< 0.05)	
Critical Value (Table ISO 5495) * Minimum correct identifications required	243		62	

* *p*-value values correspond to the exact binomial test (theoretical distribution of *p* = 0.50), calculated using SPSS v.29, following the guidelines of the ISO 5495 standard. For the critical value in the case of *N* = 451, the minimum correct identifications required was calculated using the normal approximation of ISO 5495: X = (N/2) + (1.645 x (√N/2)) = 243. The critical value of 62 correct identifications for *N* = 106 is extracted directly from the tables of the standard in its unilateral annex.

**Table 7:** Wine absorbance evolution at 420, 520 and 620 nm during bottle aging (mean and standard deviation) over time (0, 3, 6, 12, 24, 36).

Time	Abs 420 nm			Abs 520 nm			Abs 620 nm		
Months	Test (14% *v*/*v*)	Control (15% *v*/*v*)	(*p*-Value)	Test (14% *v*/*v*)	Control (15% *v*/*v*)	(*p*-Value)	Test (14% *v*/*v*)	Control (15% *v*/*v*)	(*p*-Value)
0	0.201 ± 0.000	0.206 ± 0.000	ns	0.058 ± 0.000	0.058 ± 0.000	ns	0.025 ± 0.000	0.020 ± 0.000	ns
3	0.206 ± 0.004	0.212 ± 0.001	ns	0.057 ± 0.001	0.061 ± 0.001	ns	0.025 ± 0.001	0.024 ± 0.002	ns
6	0.211 ± 0.004	0.215 ± 0.001	ns	0.057 ± 0.005	0.059 ± 0.001	ns	0.027 ± 0.005	0.023 ± 0.002	ns
12	0.224 ± 0.004	0.227 ± 0.006	ns	0.063 ± 0.002	0.065 ± 0.004	ns	0.022 ± 0.001	0.021 ± 0.003	ns
24	0.239 ± 0.002	0.228 ± 0.002	* (0.0124)	0.071 ± 0.000	0.067 ± 0.001	ns	0.027 ± 0.002	0.024 ± 0.003	ns
36	0.252 ± 0.001	0.240 ± 0.000	** (0.0066)	0.071 ± 0.000	0.068 ± 0.001	ns	0.020 ± 0.000	0.022 ± 0.000	ns

* Asterisks indicate statistically significant differences between alcohol levels within the same wine type at each specific time point according to two-way ANOVA followed by Šidák’s multiple comparisons test (* *p* < 0.05, ** *p* < 0.01; ns: not significant, *p* ≥ 0.05).

## Data Availability

The original contributions presented in this study are included in the article/Supplementary Material. Further inquiries can be directed to the corresponding author.

## Supplementary Materials

The following supporting information can be downloaded at: https://www.mdpi.com/article/10.3390/foods15203625/s1. Table S1: Frequency of detection (%) of sensory attributes for winery A wines at 0, 3, 6, 9 and 12 months of aging. Table S2: Frequency of detection (%) of sensory attributes for winery B wines at 0, 3, 6, 9 and 12 months of aging.
