# Supplementary material for: Impact on Sensory Typicity and Consumer Acceptance of the New Regulatory Alcohol Framework for Biologically Aged Sherry Wines: An Industrial-Scale Study
Source: Foods. 2026 Oct 12;15(20):3625. doi: 10.3390/foods15203625 (PMC13704571; doi:10.3390/foods15203625)
Supplement: Supplementary file 1 [file foods-15-03625-s001.zip › foods-4566829-supplementary.pdf]

Table S1. Frequency of citation (%) of sensory attributes for winery A wines at 0, 3, 6, 9 and 12 months of aging.

Attribute (Winery A)	0control	0test	3 control	3 test	6 control	6 test	9 control	9 test	12 control	12 test
White Flowers	43	29	83	86	29	71	57	43	67	14
Apple	43	71	67	71	57	86	86	100	83	100
Citrus	0	14	0	29	0	0	14	14	0	0
Vegetable	14	14	17	14	0	0	14	0	0	14
Nuts	86	71	100	100	71	86	57	71	86	100
Yeast	100	100	100	71	86	86	100	86	86	86
SEASONED SHERRY OAK	14	43	17	29	14	29	29	29	33	29
Raisins	0	0	0	0	0	0	0	0	0	0
Caramel	0	0	0	0	0	0	0	0	0	0
Honey	0	0	0	0	0	0	0	0	0	0
Lactic	14	0	17	14	14	0	0	0	0	0

Table S2. Frequency of citation (%) of sensory attributes for winery B wines at 0, 3, 6, 9 and 12 months of aging.

Attribute (Winery B)	0control	0test	3 control	3 test	6 control	6 test	9 control	9 test	12 control	12 test
White Flowers	43	43	86	71	67	43	57	29	43	29
Apple	71	100	86	71	83	57	71	71	43	57
Citrus	29	14	29	14	0	14	0	14	0	14
Vegetable	29	0	14	43	0	14	14	14	14	14
Nuts	100	71	100	100	83	86	100	71	86	100
Yeast	100	86	71	86	83	71	86	100	100	100
SEASONED SHERRY OAK	57	29	29	43	33	29	43	43	14	14
Raisins	0	0	0	0	0	0	0	0	0	0
Caramel	0	0	0	0	0	0	0	0	0	0
Honey	0	0	0	0	0	0	0	0	0	0
Lactic	14	0	14	14	0	14	0	0	14	14
